# Large-Scale Transcriptome Analysis of Two Sugarcane Genotypes Contrasting for Lignin Content

**DOI:** 10.1371/journal.pone.0134909

**Published:** 2015-08-04

**Authors:** Renato Vicentini, Alexandra Bottcher, Michael dos Santos Brito, Adriana Brombini dos Santos, Silvana Creste, Marcos Guimarães de Andrade Landell, Igor Cesarino, Paulo Mazzafera

**Affiliations:** 1 Systems Biology Laboratory, Centre for Molecular Biology and Genetic Engineering, State University of Campinas, Campinas, SP, Brazil; 2 Department of Plant Biology, Institute of Biology, State University of Campinas, Campinas, SP, Brazil; 3 Sugarcane Center, Agronomic Institute of Campinas, Ribeirão Preto, SP, Brazil; 4 Department of Botany, Institute of Biosciences, University of São Paulo, São Paulo, SP, Brazil; ISA, PORTUGAL

## Abstract

Sugarcane is an important crop worldwide for sugar and first generation ethanol production. Recently, the residue of sugarcane mills, named bagasse, has been considered a promising lignocellulosic biomass to produce the second-generation ethanol. Lignin is a major factor limiting the use of bagasse and other plant lignocellulosic materials to produce second-generation ethanol. Lignin biosynthesis pathway is a complex network and changes in the expression of genes of this pathway have in general led to diverse and undesirable impacts on plant structure and physiology. Despite its economic importance, sugarcane genome was still not sequenced. In this study a high-throughput transcriptome evaluation of two sugarcane genotypes contrasting for lignin content was carried out. We generated a set of 85,151 transcripts of sugarcane using RNA-seq and *de novo* assembling. More than 2,000 transcripts showed differential expression between the genotypes, including several genes involved in the lignin biosynthetic pathway. This information can give valuable knowledge on the lignin biosynthesis and its interactions with other metabolic pathways in the complex sugarcane genome.

## Introduction

Sugarcane (*Saccharum* spp.) is a member of the Poaceae family with the unique property to accumulate up to 50–60% of dry weight of the mature stem as sucrose [1]. Such ability makes sugarcane economically an important for the production of sugar and bioethanol. At sugarcane mills, the juice is extracted from stalks and used for both sugar crystallization and fermentation to produce the so-called first generation ethanol [2]. So far sugarcane is among the most efficient biomass producers [3], so the industry could also benefit from the production of ethanol from the lignocellulosic feedstock represented by the bagasse, which is promptly available after sucrose extraction and is currently used for heat generation and electricity production [4]. Lignocellulose is the plant biomass composed of cellulose, hemicellulose and lignin polymers and is a renewable resource for the production of biofuels and bio-based materials [5]. However, the highly complex nature and compact structure of lignocellulose, in which cellulose microfibrils are embedded in a matrix of hemicellulose polysaccharides covalently cross-linked with the heterogenic lignin polymer, constitutes a physical barrier that limits the release of fermentable sugars, a fact known as biomass recalcitrance [6,7]. The distinctive non-linear structure of lignin, built with chemically diverse and poorly reactive linkages and a variety of monomer units, makes this phenolic polymer the major plant cell wall component responsible for biomass recalcitrance [8–10]. Consequently, biomass-to-biofuel conversion requires costly and harsh pre-treatments to remove lignin and allow the access to polysaccharides for saccharification [11]. Genetic engineering of bioenergy crops has emerged as an alternative to optimize this process, with research efforts aiming to produce plants that either accumulate less lignin or produce lignins that are more amenable to chemical degradation [12,13]. While drastic reductions in lignin deposition normally impacts plant growth and development and consequently results in yield penalties [14], even large shifts in lignin composition are generally well tolerated without negative effects on plant development and morphology [15].

Despite the recent advances in our understanding of lignin biosynthesis, many aspects remain to be explored in terms of gene expression [16], the regulation of the pathway [17,18] and lignin polymerization, in which peroxidases and laccases play an important role [19,20]. For example, despite the fact that most researchers thought that the lignin biosynthetic pathway had been fully elucidated over a decade ago, a new biosynthetic enzyme called caffeoyl shikimate esterase (CSE) was recently discovered in Arabidopsis [21]. In addition, recently the flavonoid tricin was identified as an important component of the cell wall and suggested to be a true monomer, acting as a nucleation site for lignification in monocots [22].

The information on lignin metabolism in sugarcane is still very scarce and only a few genes have been identified from large scale transcription profiling [23,24]. Only very recently, the first comprehensive study on lignin biosynthesis in sugarcane was published, reporting histological, biochemical, and transcriptional data derived from two sugarcane genotypes with contrasting lignin contents [25].

Despite of sugarcane’s economic importance, its highly complex polyploid genome has not been sequenced so far [26]. Because possibly more than five thousand sugarcane genes remain undiscovered [27] there is a need for new sequencing efforts of sugarcane transcriptomes derived from several tissues and treatments, in order to increase the basic set of sequence information and further assist biotechnological strategies. Emerging genomic technologies, such as next generation sequencing, will allow more efficient trancriptomic studies and will help in laying a platform for sugarcane genomics [28].

Here, we used RNA-seq analysis to compare the transcript profiles of developing internodes of two sugarcane genotypes contrasting for lignin content. We present an overview of the transcriptional profile of the fifth internode of sugarcane stem, and a set of more than 2,000 transcripts that shows differential expression between the contrasting genotypes.

## Materials and Methods

### Plant material, RNA extraction, library construction and Illumina sequencing

The sugarcane genotypes IACSP04-065 and IACSP04-627, contrasting for lignin content, were used in this study. The two genotypes belong to a F1 segregating progeny produced by a breeding program developed at the Sugarcane Center of Agronomic Institute of Campinas (IAC). The genotypes were evaluated for agronomic traits such as sucrose, fiber and lignin content during a three years period. Lignin content was indirectly obtained from the determination of acid and neutral detergent fibers [29]. Lignin averaged on a dry weight basis 4.32% in genotype IACSP04-065 and 8,12% in genotype IACSP04-627. Mature stalks of non-flowering plants cultivated in the field were used to obtain the setts for both cultivars. Setts were allowed to grow in the greenhouse for 9 months without light or temperature control, in containers (1 m^3^) filled with a mixture of sand and a commercial organic soil (1:1, v/v). The 5^th^ internode of each genotype was harvested and immediately separated in central (pith) and peripheral (rind) regions, and then frozen. Total RNA was extracted from these tissues using a CTAB protocol [30]. Prior to the preparation of the RNA library, the integrity and quantity of isolated RNA were assessed on Bioanalyser (Agilent Technologies). To avoid underrepresentation of transcripts that show low abundance in the pith, total RNA were mixed (1:1) and used in cDNA synthesis. Single-end Illumina mRNA libraries were generated from total RNA following the manufacturer’s instructions (Illumina Inc, San Diego, CA). The libraries were sequenced on Illumina HiSeq2000 at Fasteris SA (Fasteris Inc, Geneva, Switzerland). Raw reads (100 bp length) were retrieved in a FASTQ format.

### Data processing, de novo assembly and expression analysis

The raw reads were cleaned by removing low-quality sequences (Phred quality score < Q20) and sequences with less than 70 bp using the FASTX-Toolkit (FASTQ/A short-reads pre-processing tools, Cold Spring Harbor Laboratory). The output from the pre-processing step, a total of 22,900,400 high-quality reads, was *de novo* assembled using the Velvet/Oases suite [31,32] to generate sugarcane transcripts. Assembly results were evaluated using different hash lengths (k = 21, 23, 27, 29 and 47), and the optimal assembly of reads had hash length equal to 29. Evaluated parameters included the total number of obtained transcripts, distribution of transcripts lengths, N50 value, and mainly the results of phylogenetic analysis of previously selected genes from lignin biosynthesis pathway, as described below. GENE-counter software [33] was used to quantify gene expression, in which reads from each library were mapped against the performed assembly. Finally, the differential expression analysis were conducted with the Bioconductor edgeR package [34], with a p-value ≤ 1e^-4^. The raw reads were deposited in the National Center for Biotechnology Information (NCBI) under accession number SRP056824.

### Orthology relationship, MapMan annotation, clustering and enrichment analysis

The orthology relationship analysis was performed as described by Bottcher *et al*. [25]. Briefly, a blastx search against proteins previously shown to be involved in lignin biosynthesis was performed (e-value cutoff ≤ e^-5^) and the selected transcripts were subsequently used in a new blastx alignment including sequences from representative species of the Viridiplantae (*Arabidopsis thaliana*, *Populus trichocarpa*, *Oryza sativa*, *Sorghum bicolor*, *Selaginella moellendorffii* and *Physcomitrella patens*), whose output was further used as the data set to phylogenetic analyses [25]. The coding-sequences of the selected transcripts were deduced from the amino acids alignment with the blastx best-match hit and then aligned with the 40 first blastx hits by MAFFT [35]. The phylogenetic relationship of the aligned protein sequences was then inferred by maximum-likelihood using PhyML [36]. This process allowed identifying the putative orthologous sugarcane lignin gene for each selected transcript. Functional annotation of the transcripts was done with MapMan categories [37] using blastx (e-value ≤ 10^−5^) against *Arabidopsis thaliana* proteins. The clustering analysis of transcriptional expression profile was conducted in R package (http://www.r-project.org) using the K-means clustering algorithms, with best results obtained with 100 clusters. In a second step, the clusters were tested for MapMan terms enrichment with Fisher’s exact test (p-value < 0.05), using the PageMan application [38]. The gene test sets consisted of all transcripts in each cluster, and reference set is the whole MapMan annotation of sugarcane transcriptome described above. The Self-organizing maps (SOM) were obtained using the Flora software, http://www-microarrays.u-strasbg.fr [39].

## Results and Discussion

In a previous study, we performed a characterization of lignin deposition during sugarcane stem development, using histological, biochemical, and transcriptional data derived from two sugarcane genotypes with contrasting lignin contents [25]. Putative lignin genes were identified and their spatiotemporal expression patterns were evaluated using quantitative RT-PCR in an attempt to identify the gene family members that are likely involved in constitutive lignification in sugarcane. Here, we compared the whole transcriptome of developing internodes of two contrasting sugarcane genotypes from the same segregating progeny using next-generation sequence. Lignin content of genotype IACSP04-065 was 4.32%, whereas IACSP04-627 showed 8.12% lignin. The aim was to evaluate large-scale differences in gene expression in a plant tissue undergoing active lignification between the genotypes contrasting to lignin deposition.

### De novo assembly and quantification of the sugarcane transcriptome

Normalized cDNA libraries from a developing internode of both sugarcane genotypes were constructed and sequenced using Illumina HSeq2000 platform, generating around 28 million single-end 100 bp long reads. The pre-processing step removed 18% of the raw data, in which low-quality sequences with ambiguous bases and reads with less than 70 bp were discarded, resulting in a set of 22,900,400 high-quality reads (10,555,189 from IACSP04-627 library, and 12,345,211 from IACSP04-065). The high-quality reads were *de novo* assembled and resulted in a set of 85,151 transcripts of sugarcane. This data set can be accessed in FASTA format in the S1 File and it includes all transcripts isoforms of 51,068 assembled unigenes. These unigenes have a mean length of 987 bp and a N50 equal to 1,385 bp. In total, there were 8,315 (16%) unigenes with size between 500 bp and 1,000 bp, and 7,679 (15%) unigenes between 1 kb and 3 kb. The assembled transcripts were functional annotated with MapMan categories, using blastx against *Arabidopsis thaliana* proteins. Subsequently, the expression levels of all transcripts in IACSP04-627 and IACSP04-065 genotypes were inferred by mapping reads from each library against the performed assembly (S2 File).

### Identification and expression quantification of genes associated with phenylpropanoid biosynthesis

**Fig 1:**
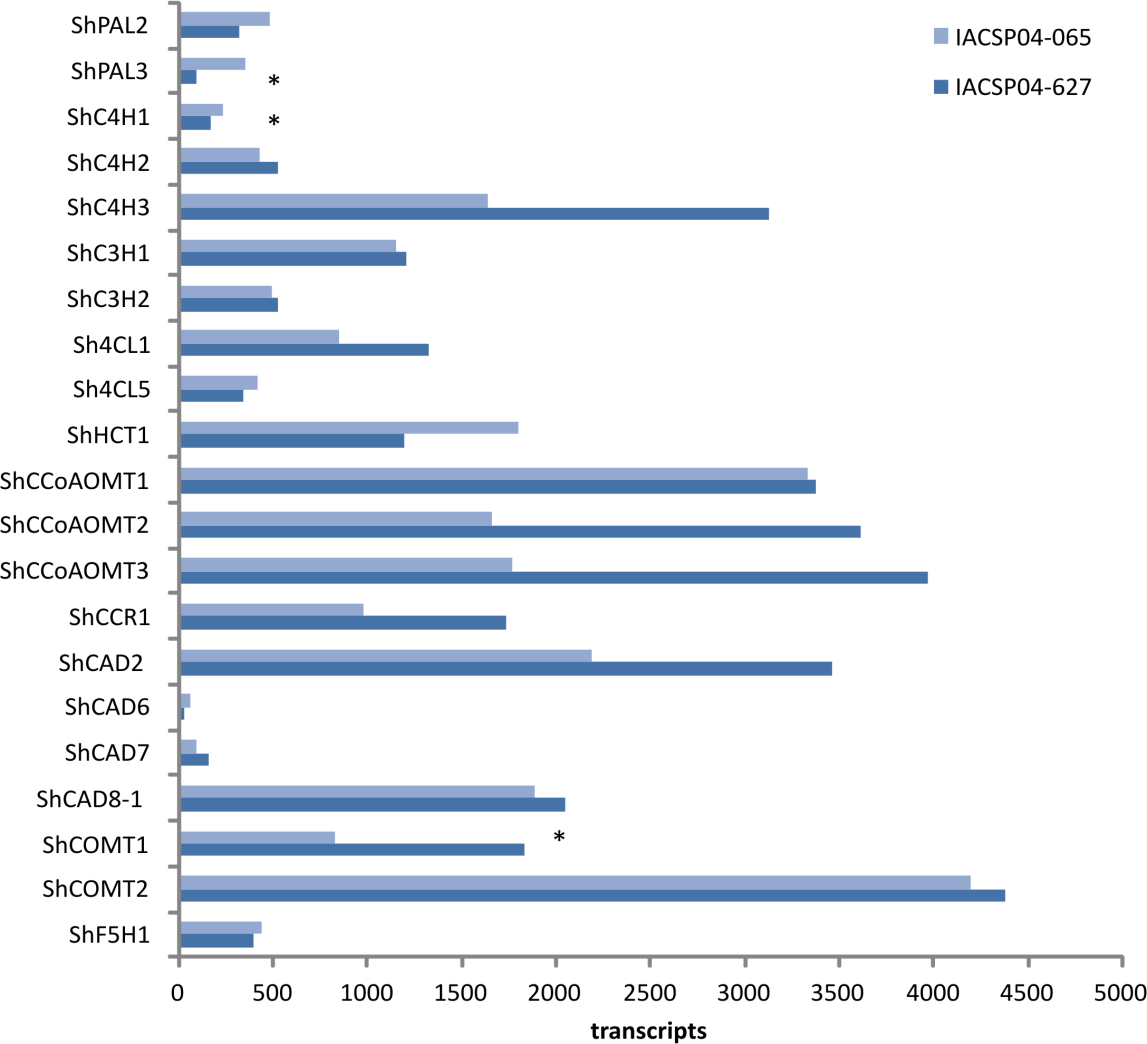
Transcript levels of lignin genes in the contrasting sugarcane genotypes. (*) indicates transcripts that are significantly (p-value ≤ 1e^-4)^ different in abundance between the two sugarcane genotypes.

The RNA-seq data were used to quantify and study sugarcane genome-wide changes in gene expression related with the phenylpropanoid biosynthesis, in particular the lignin biosynthetic pathway. Applying the previously described orthology workflow [25] it was possible to identify 21 sugarcane transcripts that encode for enzymes involved in lignin biosynthesis (Fig 1), including 2 PALs (EC 4.3.1.5), 3 C4Hs (EC 1.14.13.11), 2 4CLs (EC 6.2.1.12), 1 HCT (EC 2.3.1.133), 2 C3Hs (EC 1.14.13.36), 3 CCoAOMTs (EC 2.1.1.104), 1 CCR (EC 1.2.1.44), 2 COMTs (EC 2.1.1.68), 1 F5H, and 4 CADs (EC 1.1.1.195). The total number of identified phenylpropanoid-monolignol transcripts represents the members of lignin gene families expressed in sugarcane stems, but most likely does not represent the total number of lignin genes present in the highly polyploidy sugarcane genome. Accordingly, the total number of lignin genes present in closely related species is significantly higher. Xu and colleagues [40] performed a comparative genome analysis of lignin biosynthesis gene families across the plant kingdom, including representative species from Bryophytes, Lycophytes, Dicot Angiosperms and Monocot Angiosperms. While *Arabidopsis* genome harbors 63 lignin genes, a total of 141, 149 and 157 genes were found for sorghum, poplar and rice, respectively. Noteworthy, the results of Xu *et al*. [40] differ significantly from the data previously published by Raes *et al*. [41] and Shi *et al*. [42], in which 34 and 95 genes were annotated as monolignol genes in *Arabidopsis* and poplar, respectively. This observation highlights the fact that different approaches applied to identify homolog genes in a given genome leads to significantly different outputs.

A novel enzymatic step involving caffeoyl shikimate esterase (CSE) was recently identified as central to the lignin biosynthetic pathway in *Arabidopsis thaliana* [21]. CSE hydrolyses caffeoyl-shikimate into caffeic acid, which in turn is used by 4CL to produce caffeoyl-CoA, bypassing the so-called second reaction performed by HCT. Orthologues of bona fide *CSE* have been found in a wide range of plant species, especially dicots such as poplar, eucalyptus, tomato and *Medicago truncatula* [21]. However, although orthologues for this gene were also found in bryophytes (*Physcomitrella patens*), lycophytes (*Selaginella moellendorfii*) and gymnosperms (*Picea abies*) [43], only a few grasses with available genomic data seem to harbor bona fide *CSE* genes in their genome. Within the Poaceae family, *CSE* genes were not found in species belonging to the Pooideae subfamily, which includes *Brachypodium*, wheat, rye, barley and oat, while rice (Oryzodeae) and switchgrass (Panicoideae) harbor 3 and 1 bona fide *CSE* genes, respectively [21,44]. The Panicoideae subfamily is one of the most economically important grass groups, since it includes important grain and bioenergy crops such as switchgrass (*Panicum virgatum*) and foxtail millet (*Setaria italic*) as members of the tribe Paniceae and maize (*Zea mays*), sorghum (*Sorghum bicolor*) and sugarcane (*Saccharum* spp.) as members of the tribe Andropogoneae [45]. Switchgrass is the only member of the Panicoideae subfamily whose genome harbors a bona fide *CSE* gene, while members of the Andropogoneae tribe seem to lack orthologues of this gene. Accordingly, transcripts coding for *CSE* were not found in the transcriptomic datasets of the sugarcane genotypes used in our study, suggesting that sugarcane also does not possess a bona fide *CSE* gene and that this enzymatic step is not important for lignin biosynthesis in this plant species. Alternatively, a phylogenetically distant *CSE*-like gene might be responsible for this enzymatic activity in plants lacking a bona fide *CSE* gene. For instance, a similar scenario has been characterized for the biosynthesis of chlorogenic acids (CGAs) in switchgrass [46]. In solanaceous plants, the enzyme hydroxycinnamoyl-CoA: quinate hydroxycinnamoyltransferase (HQT) is responsible for CGA biosynthesis, but there are no close *HQT* orthologues in switchgrass. Escamilla-Treviño and colleagues [46] demonstrated that a switchgrass HCT-like enzyme exhibits HQT activity, preferring quinic acid instead of shikimate as acyl acceptor, and is most likely responsible for CGA biosynthesis. Therefore, before any further conclusions, the role for CSE in lignification of plants other than Arabidopsis awaits experimental confirmation.

The analysis of the transcriptome data showed that, with the exception of *ShC4H3*, all the genes from the general phenylpropanoid pathway (i.e. from *PAL* to *4CL*) show relatively low expression levels in both sugarcane genotypes (Fig 1), which is expected to result in low availability of monolignols. *PAL* is known to be differentially expressed through time and does not act at capacity when the demand for lignin is relatively low, for example in young internodes [47]. Although lignification is an active process in the fifth internode of sugarcane stem, this developing internode is still considered a relatively young tissue and, therefore, shows only limited lignin deposition. In accordance with this observation, the expression levels of many of these key genes significantly increased with stem maturity, which correlates with the increase of lignin content during sugarcane stem development [25].

The transcript levels of all sugarcane *CCoAOMT* genes were conspicuously high, especially in the high-lignin genotype IACSP04-627 (Fig 1). This is in contrast to what was found in Arabidopsis, in which only one family member *AtCCoAOMT1* was highly expressed in the inflorescence stem [41]. In *Populus trichocarpa*, from six identified *CCoAOMT* genes, three showed significant expression levels in developing xylem, but *PtrCCoAOMT1* was clearly the most abundant, showing more than two-fold higher expression levels when compared to *PtrCCoAOMT2* and four-fold when compared to *PtrCCoAOMT3* [42]. Since CCoAOMT was shown, together with COMT, to be involved in methylation steps necessary for the biosynthesis of not only monolignols but also soluble phenolics like flavonoids and sinapoyl malate in Arabidopsis, we could hypothesize that the high expression levels of sugarcane *CCoAOMT* genes might also be related to the production of other phenolic compounds in sugarcane. Indeed, sugarcane stem produces an array of different phenolic compounds, as revealed by the analysis of the phenolic profiles during sugarcane stem development [25]. Among the thirty-five identified compounds, all annotated flavonoids were tricin and tricin *O*-glycosides. Interestingly, the biosynthesis of tricin occurs through the sequential methylation of its precursor tricetin, in two consecutive reactions catalyzed by the same *O*-metyltransferase that shows significant similarity to COMTs and, to a lower extent to CCoAOMTs [48].

Among the *ShCAD* genes, the high expression of *ShCAD2* and *ShCAD8-1* contrasted with the very low transcript levels of *ShCAD6* and *ShCAD7* (Fig 1). It is important to mention that *ShCAD2* is the putative orthologue of *SbCAD2* from *Sorghum bicolor*, which was recently identified as the *Brown midrib6* (*Bmr6*) gene [49]. The *bmr6* mutant is affected in the phenylpropanoid metabolism, which results in reduced lignin content and altered lignin composition in sorghum. A nonsense mutation in *SbCAD2* truncates the reading frame prior to the catalytic domains of the protein, resulting in a nonfunctional enzyme and is, consequently, responsible for the *bmr6* phenotype [50]. In addition, phylogenetic analysis showed that SbCAD2 belongs to an evolutionarily conserved group of CAD proteins involved in lignin biosynthesis [50]. The high expression levels of *ShCAD2* and its close phylogenetic relationship to the lignin-related *SbCAD2* from sorghum support a role for this gene in the constitutive lignification in sugarcane.

In accordance with previous results, S branch-specific genes showed the same discrepancy in transcript levels, with very low expression levels of *ShF5H1* and much higher for both *ShCOMT* genes (Fig 1). Lower levels of *ShF5H1* expression correlates with lower S/G ratio found in younger internodes of sugarcane. This observation is in line with the fact that syringyl units can be decreased or increased simply by down-regulation or up-regulation of F5H, respectively [51]. Several lines of evidence suggest that *F5H* expression might be differentially regulated than other lignin biosynthetic genes [52]. First, AC elements (i.e. cis-elements that determine xylem-specific expression) are present in all G-branch lignin genes in Arabidopsis, but they cannot be found in the promoter region of S-branch-specific *F5H* and *COMT* [41]. Second, *F5H* is the only lignin biosynthetic gene directly regulated by the secondary cell wall master switch SND1 in *Medicago truncatula*, while all the other genes are directly regulated by SND1 targets MYB46 and its functionally redundant pair MYB58 [17]. Third, secondary wall master switches *SND1/NST1* are exclusively expressed in fibers, a xylem cell type enriched in S-lignin, while the transcripts of master switches VND6/VND7 are only found in G-rich vessels [53–55]. In contrast to the low expression of *ShF5H1*, the high transcript levels of *ShCOMT* genes already in younger internodes might be related to the above-mentioned production of soluble phenolics such as tricin, tricin *O*-glycosides and other flavonoids.

In order to evaluate whether the difference in lignin content between the genotypes is related to differential expression of lignin-specific genes, the transcript levels for each gene was compared between the contrasting sugarcane genotypes. Surprisingly, only three genes showed differential expression: *ShPAL3* and *ShC4H1* were higher in the low-lignin genotype IACSP04-065 and *ShCOMT1* showed higher expression in the high-lignin genotype IACSP04-627 (Fig 1). When comparing two genotypes contrasting for lignin content, one would expect that most of the lignin biosynthetic genes would show increased transcript levels in the high lignin genotype. However, many studies have shown that a simple correlation between lignin content and differential expression of lignin genes is not always straightforward. *ShPAL3*, *ShC4H1* and *ShCOMT1* had their expression analyzed in different tissues of two sugarcane genotypes also differing for lignin content [25] and in general the expression of the first gene was higher in the genotype containing less lignin, while the other two genes had similar expression in both genotypes. Recently we demonstrated that in sugarcane culm, the differential lignin deposition between genotypes, tissue types and at different developmental stages is under transcriptional regulation [56].

The comparison of eleven guinea grass genotypes differing in cell wall digestibility and lignin content revealed that the expression of only two lignin genes, *C4H* and *CCoAOMT*, was increased in the high-lignin genotypes [57]. Similarly, a microarray-based approach showed that only *CCoAOMT* was up-regulated in the high-lignin genotype of *Eucalyptus grandis* when compared to low lignin genotypes [58]. Even more surprising, when RNA-seq was used to compare two alfalfa genotypes with divergent cell wall composition in stems, some lignin genes (e.g. *CAD* and *COMT*) were up-regulated while others (e.g. *PAL*) were down-regulated in the high lignin content genotype [59]. Noteworthy, while the formers encode enzymes involved in downstream steps of the pathway, the later is the entry point enzyme that directs the carbon flux into the phenylpropanoid pathway and therefore, would be more likely to control the overall lignin content in the plant.

Recent studies have demonstrated that lignin deposition is not only regulated at the transcriptional level but also at the post-transcriptional and post-translational levels and through the control of enzyme catalytic activities. In the stem differentiating xylem of *Populus trichocarpa*, the microRNA ptr-miR397a was shown to be a negative regulator of laccase genes involved in lignin polymerization. Overexpression of ptr-miR397 resulted in the down-regulation of 17 laccase genes without any effect on the transcript levels of monolignol biosynthetic genes, which led to a reduction in lignin content of up to 22% [60]. In Arabidopsis, Kelch repeat F-box (KFB) proteins physically interact with PAL isoenzymes and mediate their ubiquitination and subsequent proteolytic degradation via the 26S proteasome pathway. The expression of *KFB* genes varies among plant tissues and responds to developmental and environmental cues, which contributes to the dynamic control of PAL activity. By affecting the stability of the PAL enzymes, KFBs post-translationally regulate the levels of phenylpropanoids and lignin [61]. Finally, many studies have shown that phenylpropanoid intermediates (e.g. hydroxycinnamates and shikimate esters) are able to inhibit different steps of the pathway, working as regulators of the carbon flux towards the different branches of the phenylpropanoid-monolignol metabolic grid [62–64]. As lignification represents a non-recoverable investment of carbon and energy [65], it is understandable that the regulatory mechanism controlling lignin deposition is so complex, involving a multi-leveled feed-forward loop transcriptional network that modulates the expression of biosynthetic genes [66] and post-transcriptional and post-translational processes that fine-tune the regulatory system.

**Fig 2:**
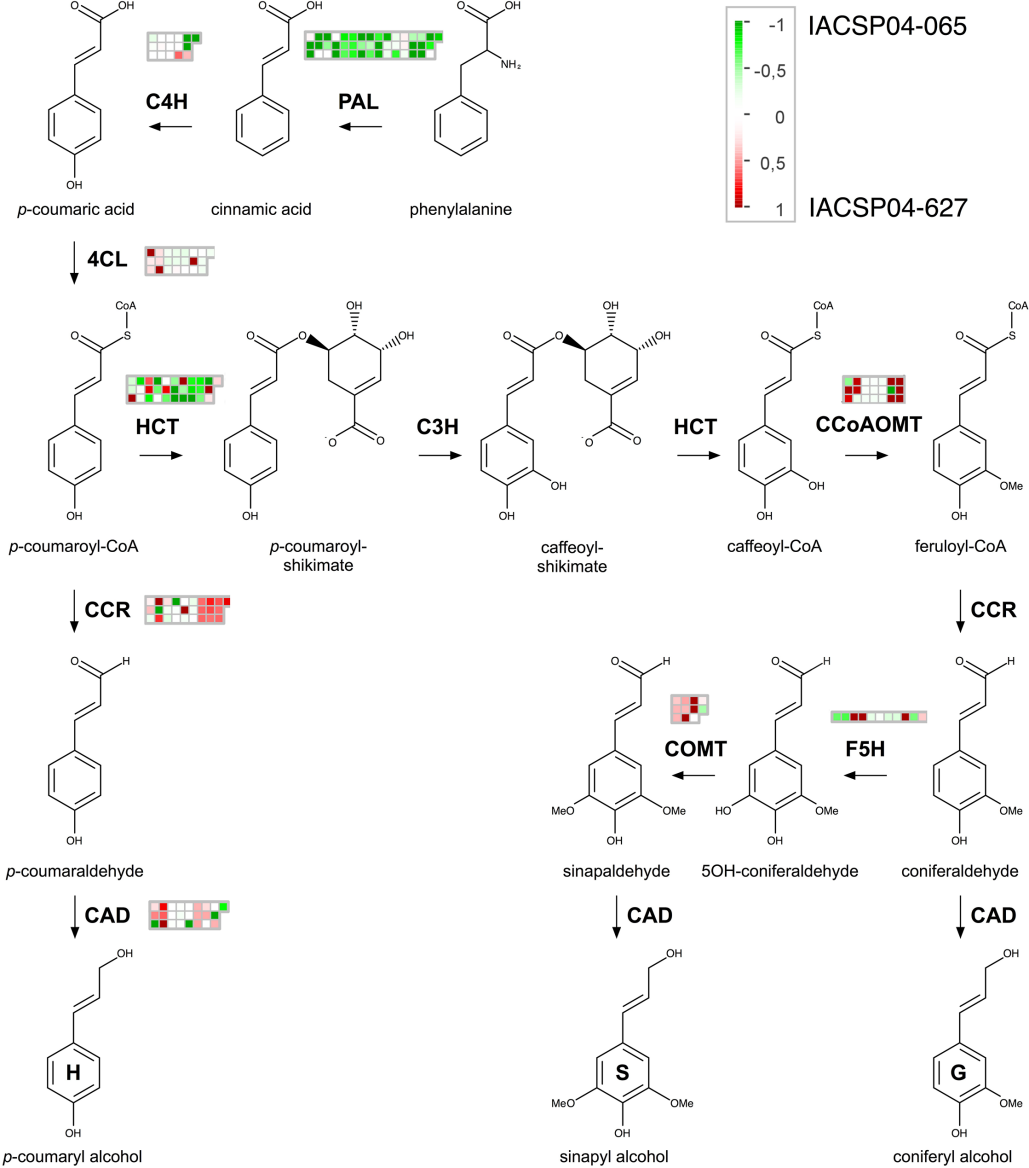
Simplified scheme of the phenylpropanoid biosynthesis pathway with genes identified in sugarcane transcriptome. Pair-wise comparisons were made between both genotypes. Each heatmap represent lignin transcript sequences identified in RNA-seq. The log2 (IACSP04-627 / IACSP04-065) expression ratio values were false color-coded using a scale of -1 to 1 and indicate the genotype that showed highest expression for each transcript. The intensity of green and red indicates the degree of down- and up-regulation of the corresponding lignin gene.

Despite of the orthology relationship previously showed, the use of high-throughput sequencing technology can also be useful to estimate the diversity of transcripts isoforms and gene families. As sugarcane is a polyploid species, polymorphisms can be generated from a different number of allelic copies present in each genotype [27]. Therefore, sugarcane cultivars are highly heterozygous, with several different alleles at each locus, and this high level of genetic complexity creates challenges during conventional and molecular breeding [67]. Some studies showed that a mutation in a single lignin biosynthesis gene can affect the expression of several other genes of the exact same biosynthetic pathway [16,68,69]. In this type of study with sugarcane it is possible to detect many transcripts that show high homology to functional genes. The MapMan analysis of sugarcane transcripts related with the phenylpropanoid pathway (Fig 2) shows transcripts of genes that encode the same enzyme and had different expression pattern between the two genotypes (higher or lower expression levels). However some gene families show a clear pattern. For instance, all transcripts of genes encoding *ShPAL* genes showed lower expression levels (or no differential expression) in the high lignin content genotype IACSP04-627 (Fig 1). *ShHCT* genes also showed strong divergent expression patterns between both genotypes, with some transcripts more expressed in the high-lignin genotype (in red) and others showed higher expression in the low-lignin genotype (in green).

### Whole sugarcane transcriptome differential expression and functional enrichment analysis

**Fig 3:**
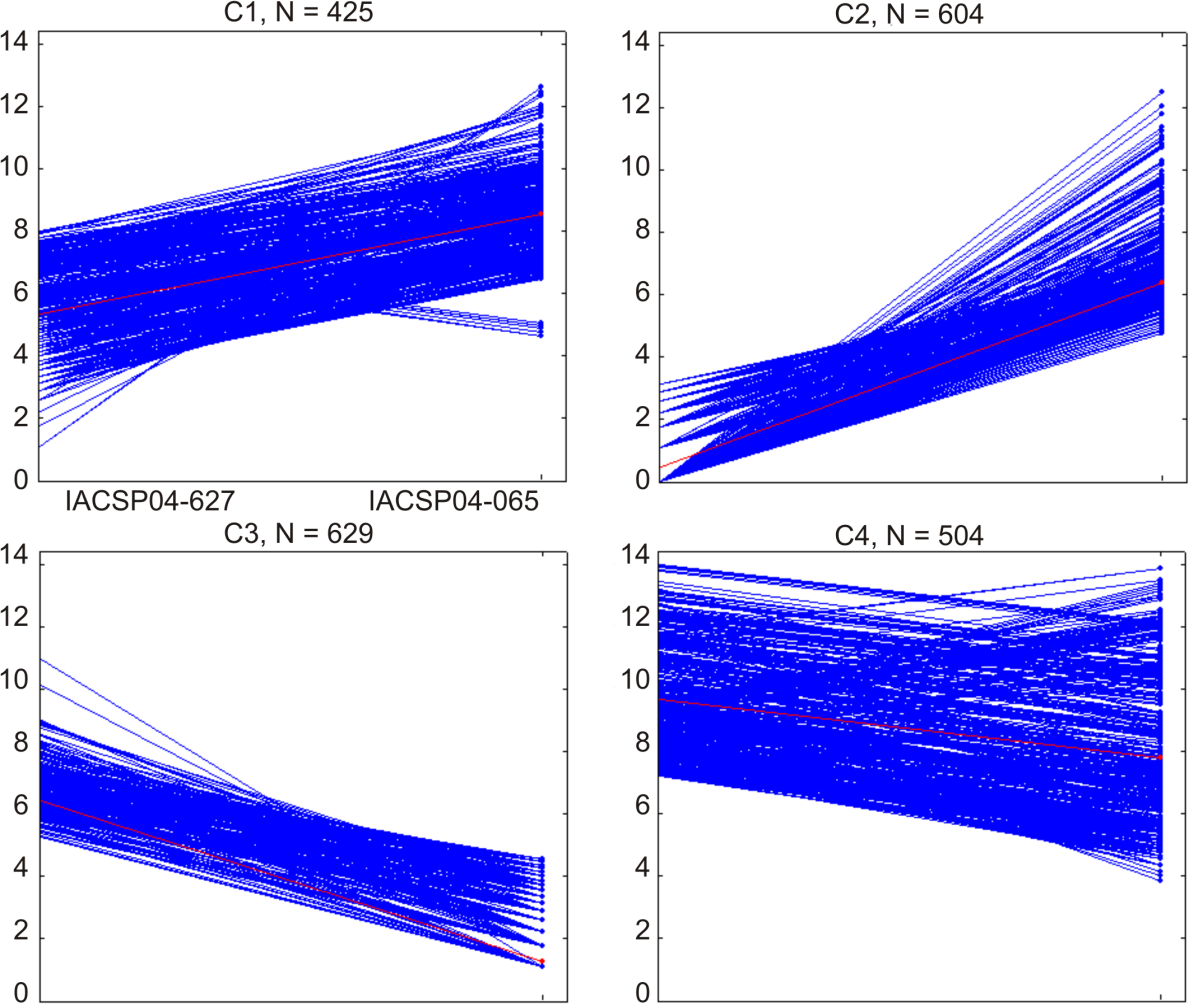
Expression pattern of 2,162 differentially expressed transcripts between genotypes IACSP04-627 (high-lignin) and IACSP04-065 (low-lignin). The number (N) indicates the amount of transcripts presented in each cluster. The y-axis shows the reads count, on log2 scale, of each transcript. Cluster 1 (C1) represents the profile of transcripts that increased in IACSP04-065 sugarcane genotype. Cluster 2 shows the profile of transcripts that had a minimal abundance in IACSP04-627 and high abundance in IACSP04-065. Cluster 3 and Cluster 4 shows an inverted pattern, where transcripts are more abundant in IACSP04-627 genotype.

Besides the expression profile of phenylpropanoid biosynthetic genes, the transcriptome obtained from both contrasting genotypes was also analyzed for the whole differential expression. This analysis resulted in 2,162 differentially expressed transcripts (S3 File) that were clustered by self-organizing maps (Fig 3). This clustering analysis shows four different expression patterns for these 2,162 differentially expressed transcripts. The Cluster 1 (C1, with 425 sugarcane transcripts) represents the profile of transcripts with higher expression levels in the IACSP04-065 sugarcane genotype. Cluster 2 (C2, n = 604) has the profile of transcripts that had a minimal abundance in IACSP04-627. Cluster 3 (C3, n = 629) and Cluster 4 (C4, n = 504) shows the opposite of C1 and C2, where transcripts are more abundant in the IACSP04-627 genotype.

**Fig 4:**
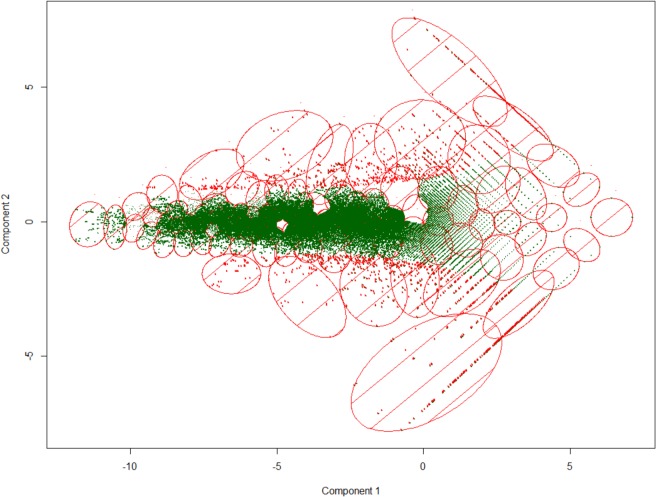
The K-means clusters of the identified sugarcane transcripts. Each dot represents a transcript. In case of differential expressed transcripts (n = 2,162) the dot is indicated in red. Transcripts within the same ellipse belong to the same cluster (100 clusters in total), according to K-means analysis. Axis indicates the first two principal components that explain the transcription expression variability. Several clusters have a significant enrichment for certain MapMan classes (according to PageMan), how showed in Fig 5 and Table 1.

**Fig 5:**
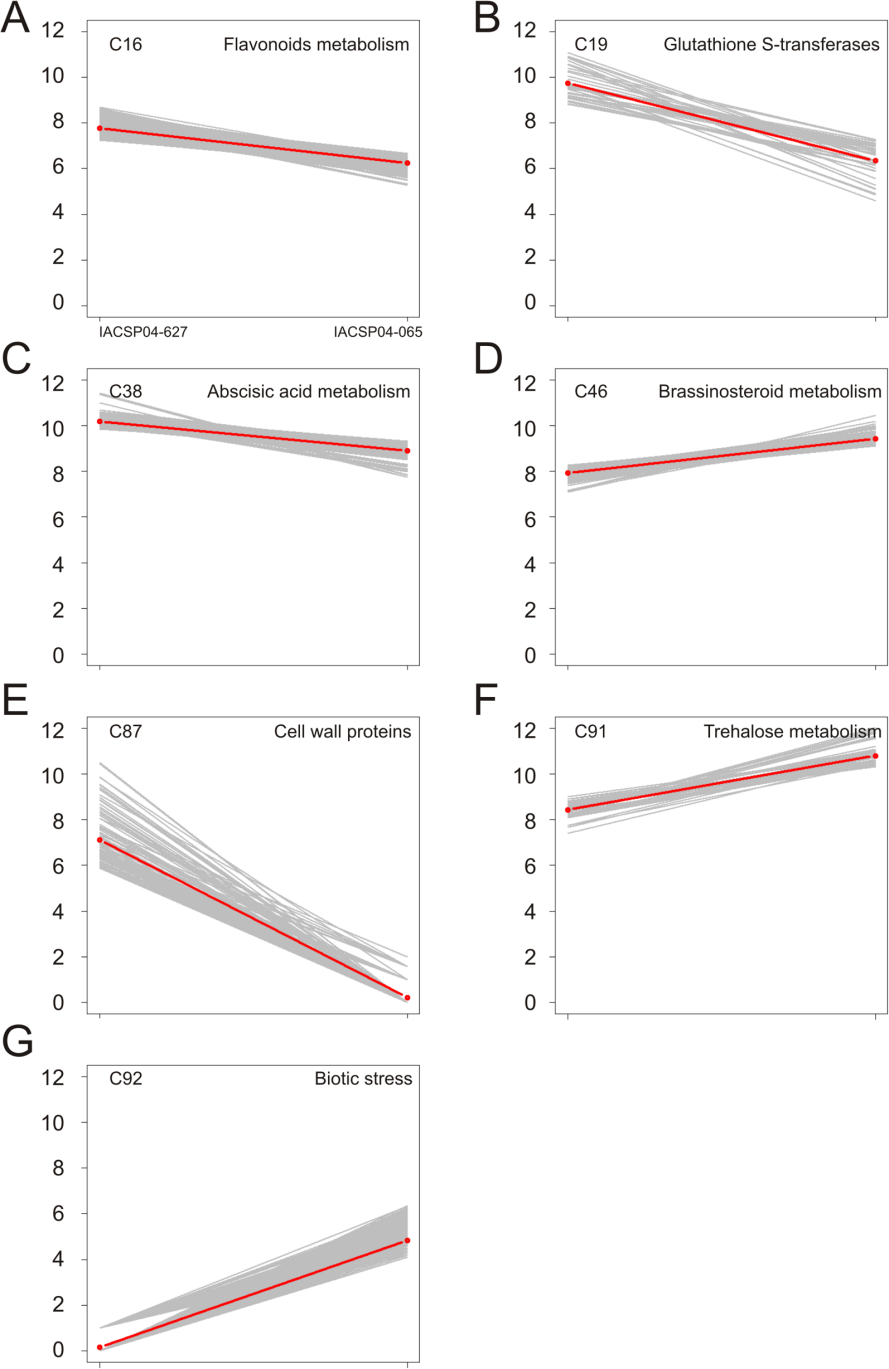
Expression profile of transcripts in selected clusters from the K-means cluster analysis. The y-axis shows the reads count, on log2 scale, of each transcript. All clusters contain differentially expressed genes and are enriched for MapMan terms. The major MapMan term enrichment for each cluster is indicated above each plot. Red line shows the mean of data plotted.

A general overview of the whole sugarcane transcriptome of both genotypes allowed the establishment of 100 clusters that share genes with similar expression pattern (Fig 4), in which several clusters have a significant enrichment for certain MapMan classes (according to PageMan) [38]. S4 File shows the K-means cluster associated with each transcript, while S5 File presents the MapMan term enriched in each cluster. The MapMan system was used to annotate the whole sugarcane transcriptome dataset (S6 File). This analysis results in clusters (Fig 5) enriched for flavonoids metabolism, glutathione-S-transferases (GSTs), abscisic acid and brassinosteroids metabolism, cell wall proteins, trehalose metabolism and response to biotic stress.

Transcripts involved with flavonoids metabolism (Fig 5A) showed a very similar expression pattern between both genotypes, with a higher expression in IACSP04-627 (high-lignin) than IACSP04-065 (low-lignin). Thus, although we have not observed a general and significant differential expression of lignin genes, the expression pattern of flavonoid-related genes and the higher lignin content in genotype IACSP04-627 suggest that both phenolic routes might be transcriptionally coordinated. Table 1 shows the transcripts that are present in the cluster enriched for flavonoid metabolism, as derived from the K-means analysis, in which all the transcripts are more expressed in the IACSP04-627 genotype.

**Table 1:** Transcripts of the cluster enriched for flavonoid metabolism (C16), as derived from the K-means analysis.

Transcript	Annotation	IACSP04-627	IACSP04-065	Transcript	Annotation	IACSP04-627	IACSP04-065
**CHO metabolism**				**Phenylpropanoids and Flavonoids**			
Locus5751.3	AGPase	204	98	Locus15211.1	Phenylpropanoids	202	85
Locus10694	TPP	174	76	Locus20492.1	CCR1	168	63
**Glyceraldehyde 3-phosphate dehydrogenase**				Locus14733.2	Indole synthesis	159	75
Locus3160	GAP-DH	235	87	Locus737 (*)	Chalcone isomerase	226	71
Locus14750	GAP-DH	197	100	Locus5932 (*)	Flavonoid 3-monooxygenase	198	66
Locus10516.3 (*)	glucose-6-phosphate isomerase	344	90	Locus3068.1 (*)	Flavonols	352	86
**Cellulase and hemicellulose synthesis**				Locus9247 (*)	Isoflavonols	344	49
Locus9999.1 (*)	COBRA	189	49	**Hormone metabolism**			
Locus10531	Glucuronoxylan	184	94	Locus3016.1	Induced-regulated-responsive-activated	154	66
**Cell wall proteins**				Locus5262 (*)	Sterols	254	39
Locus12861.6	AGP	212	68	Locus7968.1	1-aminocyclopropane-1-carboxylate oxidase	207	99
Locus11155.3	AGP	162	77	Locus665.2	Ethylene transduction	151	69
Locus9740 (*)	LRR	248	65	Locus551	ent-kaurenoic acid hydroxylase/oxygenase	187	74
Locus2657 (*)	LRR	289	80	**Biotic stress**			
Locus327.7	RGP	250	86	Locus17276	PR proteins	174	85
Locus12743.2	Mannan-xylose-arabinose-fucose	255	93	**Abiotic stress**			
Locus10669 (*)	Pectate lyases	352	52	Locus2899	Cold responsive	171	69
Locus120.9	Cell wall modification	239	101	**Peroxidases**			
**Lipid metabolism**				Locus7525.1	Peroxidase	171	85
Locus13941	Phospholipid synthesis	200	78	Locus2695	Peroxidase	209	93
Locus8761.2 (*)	Lipid transfer proteins	322	52	Locus2899.2	Peroxidase	172	67
**Amino acid metabolism**				**Signaling**			
Locus6259.2	Homocysteine S-Methyltransf.	206	86	Locus1680.1	LRR V	224	99
Locus8296.1 (*)	Glycine metabolism	250	40	Locus984.8 (*)	DUF26	284	45
Locus8258.2 (*)	Tyrosine metabolism	394	59	Locus665.4	Proline extensin like	178	57
**S-assimilation**				Locus13701.1	Calcium	161	66
Locus8310.1	Sulfite oxidase	171	84	Locus8769	Calcium	167	59
**Metal handling**				Locus5683	Calcium	186	81
Locus18021	Binding, chelation and storage protein	170	75	Locus13304.1	G-protein	254	86
				Locus8917.1 (*)	Light signalling	261	76

Their expression over the two sugarcane genotypes is given. All transcripts were more expressed in IACSP04-627 samples.

(*) indicate differential expressed transcripts (p-value < 0.05). When several isoforms were identified table shows the transcript mean expression in each genotype.

The IACSP04-627 genotype showed higher levels of transcripts related with GSTs (Fig 5B), while genotype IACSP04-065 showed higher expression of genes involved with biotic stress (Fig 5G). GSTs catalyze the detoxification of xenobiotics by their conjugation with the reduced form of glutathione (γ-glutamil-cistenil-glicine; GSH) and an electrophilic substrate, such as reactive oxygen species (ROS) [70,71]. GSTs are also thought to play major roles in oxidative stress metabolism induced by abiotic and biotic stresses [72].

The transcripts of genes involved with trehalose metabolism (Fig 5F) showed higher levels in genotype IACSP04-065. Trehalose metabolism is apparently associated with the ability to resist to abiotic stresses, in particular desiccation, although there are indications that it may also play a role in biotic stresses induced by microorganisms [73]. In this regard, the higher expression of transcripts of trehalose metabolism (Fig 5F) in the genotype IACSP04-065 is in agreement with the higher expression of biotic stress-related genes in the same genotype (Fig 5G). Further experiments would confirm the capacity of this genotype to better cope with different stress conditions.

## Conclusions

Most of the current knowledge about lignin metabolism was obtained with studies in dicotyledonous plants like Arabidopsis and poplar, but the mechanisms underlying several aspects of lignin biology such as biosynthesis, polymerization and regulation are not necessarily conserved among all vascular plants [74]. For instance, despite of its economical importance for bioethanol production, genetic information on lignin metabolism in sugarcane is limited. Only recently, the first lignin-specific gene was characterized in sugarcane using reverse genetics, by down-regulating the expression of a *COMT* [75]. In addition, evidence for the involvement of a specific laccase in the lignification process in sugarcane was obtained by the complementation of *lac17* mutant in Arabidopsis with a sugarcane laccase gene, *SofLAC* [19,76]. Despite these recent advances, there is still much to be explored and determined in terms of gene expression and pathway regulation of lignin biosynthesis in sugarcane. In this regard, the data reported here provide a comprehensive resource for lignin studies in sugarcane. In addition, several clusters of co-expressed genes potentially involved in flavonoids and carbohydrate metabolism, stress response, hormone metabolism were identified between contrasting lignin content genotypes (Fig 5 and Table 1). In general, most of the lignin genes were less expressed on the low lignin content genotype IACSP04-065, with exception for differentially expressed PAL3 and C4H1 genes. Finally, the expression profile obtained (Figs 1 and 2) for both contrasting lignin content varieties should indicate targets for further biotechnological approaches.

## Supporting Information

S1 File Transcripts sequences of the 85,151 sugarcane transcripts in FASTA format. (ZIP)

S2 File Expression levels of all transcripts in IACSP04-627 and IACSP04-065 genotypes. (TXT)

S3 File Expression levels, in log2 scale, of the 2,162 differentially expressed transcripts. (TXT)

S4 File 100 K-means clusters associated with 85,151 sugarcane transcripts. (TXT)

S5 File MapMan term enriched identified for each K-means cluster. (TXT)

S6 File MapMan annotation for the whole (85,151) sugarcane transcripts dataset. (TXT)
