# Correction: Large-Scale Transcriptome Analysis of Two Sugarcane Genotypes Contrasting for Lignin Content

**DOI:** 10.1371/journal.pone.0137698

**Published:** 2015-09-01

**Authors:** Renato Vicentini, Alexandra Bottcher, Michael dos Santos Brito, Adriana Brombini dos Santos, Silvana Creste, Marcos Guimarães de Andrade Landell, Igor Cesarino, Paulo Mazzafera

## Notice of Republication

This article was republished on August 21, 2015, due to the release of confidential or copyrighted material. Please download this article again to view the correct version.
